# Beta-globin Gene Mutations in Turkish Children with Beta-Thalassemia: Results from a Single Center Study

**DOI:** 10.4084/MJHID.2013.055

**Published:** 2013-09-02

**Authors:** Ali Fettah, Cengiz Bayram, Nese Yarali, Pamir Isik, Abdurrahman Kara, Vildan Culha, Bahattin Tunc

**Affiliations:** Ankara Children’s Hematology and Oncology Research Hospital, Department of Hematology. Ankara, Turkey

## Abstract

**Introduction:**

The beta thalassemias are common genetic disorders in Turkey and in this retrospective study our aim was to evaluate β-globin chain mutations and the phenotypic severity of β-thalassemia patients followed-up in our hospital, a tertiary center which serves patients from all regions of Turkey.

**Materials and Methods:**

106 pediatric patients were analysed for β-globin gene mutations by using DNA analysis. Patients were classified as having β-thalassemia major or β-thalassemia intermedia based on age at diagnosis, transfusion frequency and lowest hemoglobin concentration in between transfusions.

**Results:**

There were 106 patients (52.8% female and 47.2% male) with a mean age of 11.2±5 years (1.6 – 22.3 years). Eighty-four (79.2%) patients had β-thalassemia major, whereas the remaining 22 patients (20.8%) were identified as having β-thalassemia intermedia. Overall, 18 different mutations were detected on 212 alleles. The most frequently encountered mutation was IVS I.110 (G>A) (35.3%), followed by Codon 8 del-AA (10.4%), IVS II.1 (G>A) (8%), IVS I.1 (G>A) (7.5%), Codon 39 (C>T) (7.1%) and Codon 5 (−CT) (6.6%), which made up 79.4% of observed mutations. According to present results, IVS I.110 (G>AA) was the most frequent mutation observed in this study, as in other results from Turkey. Evaluation of β-thalassemia mutations in 106 patients with 212 alleles, revealed the presence of homozygous mutation in 85 patients (80.2%) and compound heterozygous mutation in 21 patients (19.8%). The mutations detected in patients with homozygous mutation were IVS I.110 (G>A) (38.8%), Codon 8 del –AA (11.8%), IVS II.1 (G>A) (8.2%) and IVS I.1 (G>A) (8.2%). Observed mutations in the compound heterozygotes were Codon 39 (C>T)/Codon 41–42 (−CTTT) (14.3%), IVS I.110 (G>A)/Codon 39(C>T) (14.3%), IVS I.110 (G>A)/Codon 44(−C) (14.3%), and IVS II.745 (C>G)/5′UTR + 22 (G>A) (9.5%).

**Conclusion:**

Our hospital is a tertiary referral center that provides care to patients from all over the country, and thus the distribution of mutations observed in the current study is significant in term of representing that of the country as a whole.

## Introduction

The beta thalassemias is a group of hereditary disorders with autosomal recessive inheritance characterized by the presence of defective synthesis of the β globin chain, an integral component of the hemoglobin molecule, resulting in either partial synthesis (β+) or complete absence (β0).^1^,^2^ The clinical and hematological spectrum of β-thalassemia ranges from a silent or thalassemia carrier state, to clinically manifest conditions such as β-thalassemia major and β-thalassemia intermedia.^1^,^3^

β-thalassemia is a worldwide condition with an overall carrier frequency of 2–25%, with cases mostly reported from the Mediterranean region, including Turkey, the Middle East, Central Asia, India, the Far East and Africa.^4^ Although the mean carrier frequency of β-thalassemia in Turkey is 2.1%, rates as high as 10% have been reported from certain regions of the country.^5^,^6^

On a molecular level, β-thalassemia mutations are quite heterogeneous, with more than 300 different mutations described in the literature.^6^,^7^ To date, 40 mutations have been reported from Turkey, the most frequent 6 mutations (IVS I-110 G>A, IVS I.6 T>C, Codon 8 −AA, IVS I.1 G>A, IVS II.1 G>A, IVS II.745 C>G) compromising 70% of all reported mutations.^6^,^8^,^9^

In this study, we evaluated β-globin chain mutations and the phenotypic severity of β-thalassemia patients followed-up in our hospital, a tertiary center which serves patients from all regions of Turkey. Further we aimed to establish the correlation between β-thalassemia mutation type and clinical manifestations of patients presenting as β-thalassemia major or β-thalassemia intermedia.

## Materials and Methods

### Patient selection and study design

This retrospective study was undertaken by the department of Pediatric Hematology at Ankara Children’s Hematology and Oncology Hospital. The medical files of 111 patients diagnosed with β-thalassemia were systematically reviewed, and 106 patients with complete records were included in the study. Five patients were excluded from the study, because they continued their follow-up in another center. Information regarding age, gender and identified β-thalassemia mutation was noted for each patient.

Patients were classified as having β-thalassemia major or β-thalassemia intermedia based on age at diagnosis, transfusion frequency and lowest hemoglobin concentration in between transfusions. A clinical diagnosis of β-thalassemia intermedia was made in patients with an age at diagnosis of more than two years with a history of less than 8 transfusions per year and hemoglobin concentration nadir between transfusions of >7 g/dl. Phenotype-genotype correlation was evaluated for each β-thalassemia patient.

### Mutation analysis

Written informed consent was obtained from the parents of all participating subjects. All blood samples have been sent to a private genetic center affiliated with our hospital in 5 ml EDTA tubes for β-thalassemia mutation analyses.

DNA isolation was achieved by the salt precipitation method. The 3 exons coding for the Beta globulin gene as well as the promoter, first intron, 5′UTR and 3′UTR sequences are replicated by polymerase chain reaction (PCR) using suitable primers. This was followed by automated DNA sequencing for mutation analysis (ABI PRISM 3130 Genetic Analyzer, ABD), the results of which were interpreted using ABI DNA sequencing Analysis Software v5.2 program. The blood samples of patients who test negative for the 22 mutations evaluated were referred for full gene analysis for β-thalassemia.

## Results

The records of 106 patients (52.8% female and 47.2% male) were deemed sufficient for inclusion in the final analysis. Mean age was 11.2±5 years (1.6 – 22.3 years). Eighty-four (79.2%) patients had β-thalassemia major, whereas the remaining 22 patients (20.8%) were identified as having β-thalassemia intermedia. In all, %79,2 of the patients had consanguineous parents; %71,4 of these consanguineous marriages were between first cousins.

Overall, 18 different mutations were detected on 212 alleles. The most frequently encountered mutation was IVS I.110 (G>A) (35.3%), followed by Codon 8 del-AA (10.4%), IVS II.1 (G>A) (8%), IVS I.1 (G>A) (7.5%), Codon 39 (C>T) (7.1%) and Codon 5 (−CT) (6.6%), which made up 79.4% of observed mutations. Other less frequently detected mutations include IVS I.6 (T>C) (3.3%), 5′UTR+22 (G>A) (3.3%), IVS II.745 (C>G) (3.3%), Codon 44 (−C) (2.4%), Codon 41/42 46p (−CTTT) (2.4%), −28 A>G (1.9%), Codon 8–9 (+G) (1.9%), IVS I.5 (G>C) (1.9%), Codon 17 (A>T) (1.9%), Codon 15 (TTG>TGA) (1.4%), −30 T>A (0.9%) and IVS II.848 (C>A) (0.5%). Results of mutation analyses have been summarized in table 1.

Evaluation of β-thalassemia mutations in 106 patients with 212 alleles, revealed the presence of homozygous mutation in 85 patients (80.2%) and compound heterozygous mutation in 21 patients (19.8%). The consanguineous marriage rate for patients with homozygous mutation was 80%, while 67,1% of marriages were between first cousins. The consanguineous marriage rate for patients with heterozygous mutation was 38,1% and 14,3% of them were first cousins.

The mutations detected in patients with homozygous mutation were IVS I.110 (G>A) (38.8%), Codon 8 del –AA (11.8%), IVS II.1 (G>A) (8.2%) and IVS I.1 (G>A) (8.2%) (Table 2). Observed mutations in the compound heterozygotes were Codon 39 (C>T)/Codon 41–42 (−CTTT) (14.3%), IVS I.110 (G>A)/Codon 39(C>T) (14.3%), IVS I.110 (G>A)/Codon 44(−C) (14.3%), and IVS II.745 (C>G)/5′UTR + 22 (G>A) (9.5%) (Table 3).

The most commonly encountered mutations in patients with β-thalassemia major were IVS I.110 (G>A) homozygous (39.2%), Codon 8 del –AA homozygous (8.3%), IVS I.1 (G>A) homozygous (7.1%), Codon 5 (−CT) homozygous (7.1%) and Codon 39 (C>T) homozygous (4.9%), which made up two-thirds of detected mutations in this group. Other observed mutations are showed in table 4.

In patients with β-thalassemia intermedia, the most frequently detected beta chain mutation was IVS II.1 (G>A) homozygous (27.3%), followed by Codon 8 del –AA homozygous (%13,6), IVS I.6 (T>C) homozygous (13.6%), 5′UTR+22 (G>A) homozygous (9.1%), IVS I.1 (G>A) homozygous (4.5%), Codon 8–9 (+G) homozygous (4.5%), −30 T>A homozygous (4.5%), IVS I.110 (G>A)/IVS II. 848 (C>A) compound heterozygous (4.5%), IVS II.1 (G>A)/IVS 1.6 (T>C) compound heterozygous (4.5%), Codon 39 (C>T)/IVS 2.1 (G>A) compound heterozygous (4.5%), IVS I.110 (G>A)/5′UTR+22 G>A compound heterozygous (4.5%), IVS I.110 (G>A)/Codon 8 del –AA compound heterozygous (4.5%) (Table 5).

Out of the 10 patients who were homozygous for the Codon 8 del –AA mutation, 7 had β-thalassemia major, while 3 had a clinical picture compatible with β-thalassemia intermedia. On the other hand only 1 out of 7 patients with homozygous IVS II.1 (G>A) mutation had β-thalassemia major whereas the remaining 6 patients were diagnosed as having β-thalassemia intermedia. Unfortunately conditions that may influence clinical severity, such as the presence of α-globulin mutations or polymorphisms that increase HbF synthesis were not evaluated in this study.

## Discussion

There are more than 300 different known β-thalassemia mutations worldwide, 40 of which have also been reported from Turkey.^6^–^8^ In our study population, 18 different mutations were detected, the most frequent being IVS I.110 (G>A) (35.3%). It has been reported in other studies from Turkey, at a rate of 40%.^6^,^10^ Other mutations that were encountered include Codon 8 del-AA, IVS II.1 (G>A), IVS I.1 (G>A), Codon 39 (C>T) and Codon 5 (−CT). Overall, these 6 mutations made up 74.9% of all detected mutations. Surprisingly, the second most frequent mutation in Turkey, IVS I.6 (T>C), was only detected in 3.3% of our patients.^6^,^10^

In patients with β-thalassemia major, the most commonly encountered mutation was IVS I.110 (G>A) homozygous (39.2%) followed by Codon 8 del –AA homozygous (8.3%), IVS I.1 (G>A) homozygous (7.1%), Codon 5 (−CT) homozygous (7.1%) and Codon 39 (C>T) homozygous (4.9%), which made up 66.6% of all detected mutations. Similarly, the homozygous IVS II.1 (G>A) mutation was the most frequently detected mutation (27.3%) in patients with β-thalassemia intermedia, followed by homozygous Codon 8 del –AA (13.6%) and homozygous IVS I.6 (T>C) (13.6%). The distribution of mutations in our group of β-thalassemia major and intermedia patients is similar to that reported by Altay et. al.^10^

The frequency of the most common 12 β-thalassemia mutations reported by Başak et al. was also similar in our study confirming that our hospital is a tertiary referral center that provides care to patients from all over the country. Thus, the results observed in the current study is significant in term of representing that of the country as a whole.

## Figures and Tables

**Table 1:** The distribution of β-globin chain mutations

Mutation	n	%
IVS I.110 (G>A)	75	35,3
Codon 8 del –AA	22	10,4
IVS II.1 (G>A)	17	8
IVS I.1 (G>A)	16	7,5
Codon 39 (C>T)	15	7,1
Codon 5 (−CT)	14	6,6
		∑ 74,9
IVS I.6 (T>C)	7	3,3
5′UTR+22 G>A	7	3,3
IVS II.745 (C>G)	7	3,3
Codon 41/42 46p (−CTTT)	5	2,4
Codon 44 (−C)	5	2,4
		∑ 89,6
−28 A>G	4	1,9
Codon 8–9 (+G)	4	1,9
IVS I.5 (G>C)	4	1,9
Codon 17 (AAG>TAG)	4	1,9
Codon 15 (TTG>TGA)	3	1,4
−30 T>A	2	<1
IVS II. 848 (C>A)	1	<1
**Total**	85	100

**Table 2:** The distribution of homozygous β-thalassemia mutations

	Total		T. Major		T. Intermedia	
Mutation	n	%	n	%	n	%
IVS I.110 (G>A)	33	38,8	33	38,8	-	-
Codon 8 del –AA	10	11,8	7	8,2	3	3,6
IVS II.1 (G>A)	7	8,2	1	1,2	6	7
IVS I.1 (G>A)	7	8,2	6	7	1	1,2
Codon 5 (−CT)	6	7	6	7	-	-
Codon 39 (C>T)	4	4,7	4	4,7	-	-
IVS I.6 (T>C)	3	3,6	-	-	3	3,6
−28 A>G	2	2,4	2	2,4	-	-
5′UTR+22 G>A	2	2,4	-	-	2	2,4
Codon 8–9 (+G)	2	2,4	1	1,2	1	1,2
IVS I.5 (G>C)	2	2,4	2	2,4	-	-
Codon 17 (AAG>TAG)	2	2,4	2	2,4	-	-
−30 T>A	1	1,2	-	-	1	1,2
Codon 15 (TTG>TGA)	1	1,2	1	1,2	-	-
Codon 41/42 46p (−CTTT)	1	1,2	1	1,2	-	-
Codon 44 (−C)	1	1,2	1	1,2	-	-
IVS II.745 (C>G)	1	1,2	1	1,2	-	-
**Total**	85	100	68	80	17	20

**Table 3:** The distribution of compound heterozygous β-thalassemia mutations

		Toplam		T. Major		T. Intermedia	
Mutation 1	Mutation 2	n	%	n	%	n	%
Codon 39 (C>T)	Codon 41–42 (−CTTT)	3	14,3	3	14,3	-	
IVS I.110 (G>A)	Codon 39 (C>T)	3	14,3	3	14,3	-	
IVS I.110 (G>A)	Codon 44 (−C)	3	14,3	3	14,3	-	
IVS II.745 (C>G)	5′UTR + 22 G>A	2	9,5	2	9,5	-	
Codon 15 (TTG>TGA)	Codon 8 del –AA	1	4,8	1	4,8	-	
Codon 39 (C>T)	IVS II.1 (G>A)	1	4,8	-		1	4,8
Codon 5 (−CT)	IVS I.1 (G>A)	1	4,8	1	4,8	-	
Codon 5 (−CT)	IVS II.745 (C>G)	1	4,8	1	4,8	-	
IVS II.1 (G>A)	IVS II.745 ( C>G)	1	4,8	1	4,8	-	
IVS II.1 (G>A)	IVS I.6 (T>C)	1	4,8	-		1	4,8
IVS I.110 (G>A)	Codon 8 del –AA	1	4,8	-		1	4,8
IVS I.1 (G>A)	IVS II.745 (C>G)	1	4,8	1	4,8	-	
IVS I.110 (G>A)	5′UTR+22 G>A	1	4,8	-		1	4,8
IVS I.110 (G>A)	IVS II. 848 (C>A)	1	4,8	-		1	4,8
**Total**		21	100	16	76	5	24

**Table 4:** The distribution of mutations in patients with β-thalassemia major

Mutation	n	%
IVS I.110 (G>A) Homozygous	33	39,2
Codon 8 del –AA Homozygous	7	8,3
IVS I.1 (G>A) Homozygous	6	7,1
Codon 5 (−CT) Homozygous	6	7,1
Codon 39 (C>T) Homozygous	4	4,9
Codon 39 (C>T)/Codon 41–42 (−CTTT) Compound Heterozygous	3	3,6
IVS I.110 (G>A)/Codon 39 (C>T) Compound Heterozygous	3	3,6
IVS I.110 (G>A)/Codon 44 (−C) Compound Heterozygous	3	3,6
IVS II.745 (C>G)/5′UTR + 22 G>A Compound Heterozygous	2	2,4
−28 A>G Homozygous	2	2,4
IVS I.5 (G>C) Homozygous	2	2,4
Codon 17 (AAG>TAG) Homozygous	2	2,4
Codon 15 (TTG>TGA) Homozygous	1	1,2
Codon 41/42 46p (−CTTT) Homozygous	1	1,2
Codon 44 (−C) Homozygous	1	1,2
IVS II.745 (C>G) Homozygous	1	1,2
IVS II.1 (G>A) Homozygous	1	1,2
Codon 8–9 (+G) Homozygous	1	1,2
Codon 15 (TTG>TGA)/Codon 8 del –AA Compound Heterozygous	1	1,2
Codon 5 (−CT)/IVS I.1 (G>A) Compound Heterozygous	1	1,2
Codon 5 (−CT)/IVS II.745 (C>G) Compound Heterozygous	1	1,2
IVS II.1 (G>A)/IVS II.745 ( C>G) Compound Heterozygous	1	1,2
IVS I.1 (G>A)/IVS II.745 (C>G) Compound Heterozygous	1	1,2
**Total**	84	100

**Table 5:** The distribution of mutations in patients with β-thalassemia intermedia

Mutation	n	%
IVS II.1 (G>A) Homozygous	6	27,3
Codon 8 del –AA Homozygous	3	13,6
IVS I.6 (T>C) Homozygous	3	13,6
5′UTR+22 G>A Homozygous	2	9,1
Codon 8–9 (+G) Homozygous	1	4,5
IVS I.1 (G>A) Homozygous	1	4,5
−30 T>A Homozygous	1	4,5
IVS I.110 (G>A)/IVS II. 848 (C>A) Compound Heterozygous	1	4,5
Codon 39 (C>T)/IVS II.1 (G>A) Compound Heterozygous	1	4,5
IVS II.1 (G>A)/IVS I.6 (T>C) Compound Heterozygous	1	4,5
IVS I.110 (G>A)/5′UTR+22 G>A Compound Heterozygous	1	4,5
IVS I.110 (G>A)/Codon 8 del –AA Compound Heterozygous	1	4,5
**Total**	22	100
